# Process Development of the Copper(II)‐Catalyzed Dehydration of a Chiral Aldoxime and Rational Selection of the Co‐Substrate

**DOI:** 10.1002/open.202100230

**Published:** 2021-12-10

**Authors:** Jannis Nonnhoff, Harald Gröger

**Affiliations:** ^1^ Chair of Industrial Organic Chemistry and Biotechnology Faculty of Chemistry Bielefeld University Universitätsstrasse 25 33615 Bielefeld Germany

**Keywords:** aldoxime, copper(II) acetate, dehydration, *N*-acyl amino nitrile, nitrile

## Abstract

The access towards chiral nitriles remains crucial in the synthesis of several pharmaceuticals. One approach is based on metal‐catalyzed dehydration of chiral aldoximes, which are generated from chiral pool‐derived aldehydes as substrates, and the use of a cheap and readily available nitrile as co‐substrate and water acceptor. Dehydration of *N*‐acyl α‐amino aldoximes such as *N*‐Boc‐l‐prolinal oxime catalyzed by copper(II) acetate provides access to the corresponding *N*‐acyl α‐amino nitriles, which are substructures of the pharmaceuticals Vildagliptin and Saxagliptin. In this work, a detailed investigation of the formation of the amide as a by‐product at higher substrate loadings is performed. The amide formation depends on the electronic properties of the nitrile co‐substrate. We could identify an acceptor nitrile which completely suppressed amide formation at high substrate loadings of 0.5 m even when being used with only 2 equivalents. In detail, utilization of trichloroacetonitrile as such an acceptor nitrile enabled the synthesis of *N*‐Boc‐cyanopyrrolidine in a high yield of 92 % and with full retention of the absolute configuration.

## Graphical abstract

**Unwanted amide formation** in the copper(II)‐catalyzed dehydration of aldoxime can be reduced or completely suppressed by a high excess of acetonitrile or by choosing a more electrophilic acceptor nitrile such as trichloroacetonitrile. Carefully tuning these parameters enabled the synthesis of *N*‐Boc‐cyanopyrrolidine in a high yield of 92 %, with full retention of the absolute configuration.

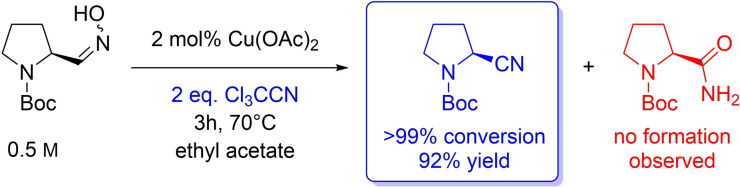

## 1 Introduction

**Figure 1:**
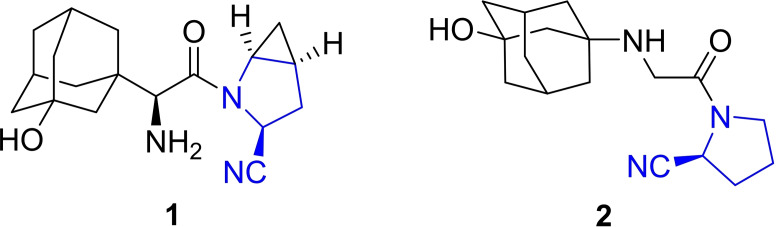
Chiral 2‐cyanopyyrolidine based DPP‐4 inhibitors.

Nitriles represent an important class of compounds for bulk and fine chemicals with a particular focus on their occurrence as chiral building blocks in pharmaceuticals.^[1]^ An example are chiral acylated *α*‐amino nitriles (Figure 1) such as Saxagliptin **1**,^[2]^ distributed by AstraZeneca/BMS, and Vildagliptin **2**,[^3^, ^4^] distributed by Novartis, with sales of over $1 billion per year. Both pharmaceuticals belong to the class of dipeptidyl peptidase‐4 (DPP‐4) inhibitors and are used in the treatment of type II diabetes. In both molecules, the (*S*)‐2‐cyanopyrrolidine structure plays a crucial role in the mechanism of action and, thus, represents a key structure.

As a result, various efforts have been made to discover new ways of synthesizing chiral nitriles and especially *α*‐amino nitriles. One example is given by the asymmetric Strecker synthesis. Starting from prochiral aldehydes, chiral *α*‐amino nitriles can be obtained using amines, cyanides and chiral catalysts.^[5]^ Despite great progress in the development of catalysts for this transformation, the use of toxic cyanides is a drawback of this approach.

The utilization of toxic cyanides in the synthesis of nitriles can be avoided by dehydration of amides and aldoximes. Such a process is used, for instance, in the synthesis of Vildagliptin **2**, and proceeds through the dehydration of *N*‐acyl protected l‐proline amide, which is easily accessible from the chiral pool reagent l‐proline amide by using a suitable Vilsmeier reagent.[^3^, ^4^] The preparation of the Vilsmeier reagent is based on the utilization of phosphoryl chloride as a substrate.

Dehydration of aldoximes represents a promising alternative for synthesizing nitriles. For the dehydration of aldoximes, several stochiometric reagents have been described, such as oxalyl chloride,^[6]^ iodine and triphenylphosphane,^[7]^ trifluoroacetic anhydride,^[8]^ Swern‐like conditions^[9]^ or chlorotropylium,^[10]^ while the aldoximes are simply obtained from their aldehydes by condensation with hydroxylamine. Some one‐pot syntheses based on the dehydration of aldoximes, obtained in situ from their aldehydes, have also been described. In particular, a dehydration with HMDS and DBU,^[11]^ the use of ethyl chlorophosphates^[12]^ or acid‐catalyzed dehydration in DMSO^[13]^ were reported.

A recent focus has been on the development of environmentally friendly and cost‐effective versions of this type of reaction, leading to the use of various metal catalysts such as copper,^[14]^ palladium,^[15]^ iron,^[16]^ cobalt and others.^[17]^ In contrast to the use of classical dehydration reagents, these catalytic versions often use other (cheap) nitriles such as acetonitrile as water acceptors, thus forming the corresponding amides as by‐products.

**Scheme 1:**
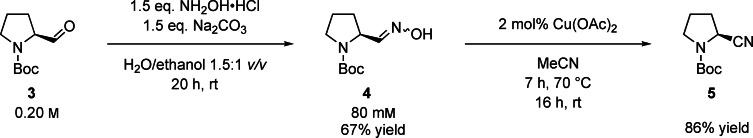
De novo synthesis of enantiomerically highly enriched *N*‐acyl amino nitrile **5** by Rommelmann et al.^[18]^

In previous studies of our group,[^3^, ^4^, ^18^] an alternative synthesis of enantiomerically highly enriched *N*‐acyl amino nitriles, based on a copper(II) acetate‐catalyzed dehydration of aldoximes, was described, using acetonitrile as water acceptor (Scheme 1). Starting from substrates derived from the chiral pool such as *N*‐Boc‐l‐prolinal **3**, condensation with hydroxylamine, thus forming Boc‐prolinal oxime **4**, and subsequent dehydration enabled a de novo‐access to *N*‐Boc‐(*S*)‐2‐cyano‐pyrroldine (**5**).[^3^, ^4^, ^18^] This process was transferred to other *N*‐acylated pyrrolidines, which could serve as intermediates in the synthesis of Vildagliptin.[^3^, ^4^, ^18^] Despite the advantages of such a cyanide‐free synthesis of acylated *N*‐amino nitriles, this access however showed disadvantages in terms of applicability on technical scale. The dehydration suffered from a low substrate concentration (80 mm), a low space‐time yield and from being a two‐step process.

During our research on scaling up this process and improving the space‐time yield of the dehydration step by raising the substrate loading of Boc‐prolinal oxime **4** to 0.5 m in the presence of acetonitrile as reagent (“acceptor nitrile”), the formation of *N*‐Boc‐proline amide **6** in significant amounts was observed, which also occurred in a developed one‐pot process.^[19]^ The reason for the formation of Boc‐proline amide **6** as a by‐product can be found when taking into account the reaction mechanism of the desired dehydration step. In particular at high substrate loading, in situ formed Boc‐cyanopyrrolidine **5** can also take over the function of the acceptor nitrile in the dehydration of unreacted aldoximes.[^20^, ^21^] Likewise, a transformation of several aldoximes and nitriles into their primary amides using copper(II) acetate in aqueous and organic media has been described in the presence of aldoximes or hydroxylamine.[^20^, ^22^] Mechanistic studies with isotopically labelled substrates show that amide formation does not occur by simple water addition. Instead, an in situ formed nitrile acts as a (formal) water acceptor in the dehydration of further aldoximes.^[23]^

## 2 Results and Discussion

**Scheme 2:**
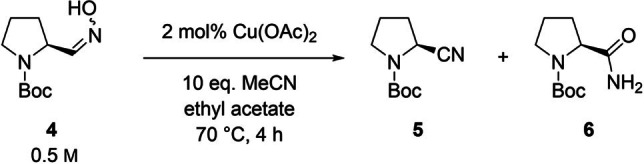
Standard synthesis of *N*‐Boc‐(*S*)‐2‐cyanopyrrolidine (**5**).

This work is focusing on an investigation of the influence of the reaction parameters on the suppression of the undesired amide formation as a side reaction. As a standard reaction, dehydration of Boc‐prolinal oxime **4** at a substrate concentration of 0.5 m, a catalyst loading of 2 mol % and the use of 10 equivalents of acetonitrile in ethyl acetate for 4 h at 70 °C was selected (Scheme 2 ).

### 2.1 Variation of Catalyst Loading and Temperature

First, the influence of the amount of copper acetate on the progress of the reaction and side product formation was studied by varying the catalyst loading between 0.5 and 5 mol % (see Supporting Information). While no difference was observed between 1 and 5 mol % with respect to the total conversion as well as to the amide formation, the total conversion at a catalyst loading of 0.5 mol % decreased to 38 % (28 % nitrile **5** and 10 % amide **6**). Thus, a catalyst loading of 2 mol % was chosen for the subsequent study of the impact of the reaction temperature on conversion and amide formation. The temperature was varied in the range of 70 °C to −20 °C using the standard reaction conditions (Table 1). The dehydration of aldoxime **4** takes place at all selected reaction temperatures, although with different rates. While the conversions at 70 °C and 50 °C are quantitative after four hours, the reaction time must be extended at lower temperatures in order to achieve full substrate consumption. It is also noteworthy that a reaction at −20 °C, without stirring, also leads to the formation of both nitrile and amide. To evaluate the effect of the temperature on amide formation, the reactions were carried out until complete conversion of the oxime was observed. When comparing the results, only a small effect of temperature can be noted, but there seems to be a tendency for a higher degree of amide formation at lower temperature. After complete conversion at 70 °C, 29 % of amide **6** are formed, in contrast to 37 % at 30 °C and 32 % at room temperature. To gain insight into the amide formation as a side reaction, a reaction without acetonitrile at room temperature and at 70 °C was also studied (Table 1, entries 9, 10). While almost no conversion of prolinal oxime **4** after 18 h was observed at room temperature, 7 % of nitrile **5** and 13 % of amide **6** were detected at 70 °C. This supports the assumption that the formation of the amide side product proceeds, at least in part, through the reaction mechanism of the aldoxime to nitrile conversion (in which the formed nitrile **5** then acts as a nitrile acceptor). At the same time, the presence of acetonitrile leads to an increased formation of amide **6** at both studied temperatures. This can be explained by the formation of the desired cyanopyrrolidine **5** in higher amount, which then acts as an intermediate in the undesired formation of the proline amide side product **6**.

**Table 1:** Screening of temperature in copper(II)‐catalysed dehydration of prolinal oxime **4**.

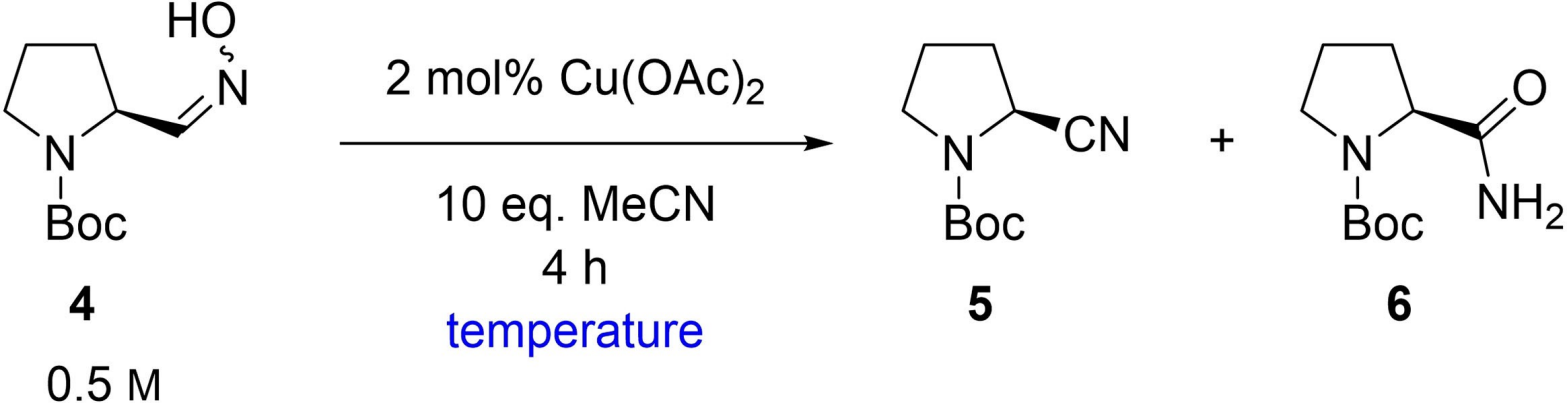					
Entry	T [°C]	Time [h]	Aldoxime **4** [%]	Nitrile **5** [%]	Amide **6** [%]
**1**	70	4	–	71	29
**2**	50	4	–	67	33
**3**	30	4	7	64	30
**4**	30	18	–	63	37
**5**	rt	4	21	63	16
**6**	rt	18	–	68	32
**7**	rt^[a]^	18	2	65	32
**8**	−20^[a]^	18	81	14	5
**9**	rt^[b]^	18	97	2	1
**10**	70^[b]^	18	80	7	13

[a] Without stirring; [b] without acetonitrile.

### 2.2 Variation of the Excess of the Acceptor Nitrile

Since an influence of the acetonitrile on conversion and side‐product formation could be observed, the used equivalents were subsequently varied in a standard reaction at 70 °C. In detail, the number of equivalents was varied between 235 and 10, which in most cases has also been associated with a change in substrate concentration (Table 2). By increasing the amount of acetonitrile, the formation of side product **6** in the dehydration of prolinal oxime **4** can be reduced or almost suppressed. For example, an increase from 10 to 38 equivalents of acetonitrile in a reaction at 0.5 m substrate concentration leads to a significant decrease of the formation of side product **6** (26 % to 11 %, entries 8, 9). By successively increasing the number of equivalents of acetonitrile up to 235, which corresponds to a substrate concentration of 80 mm of **4** in pure acetonitrile, led to a further decrease of formation of the undesired amide **6** to only 1 % at a complete conversion of the aldoxime after 4 h (entry 1).

**Table 2:** Influence of acetonitrile equivalents and substrate loading on copper(II)‐catalyzed dehydration of prolinal oxime **4**.

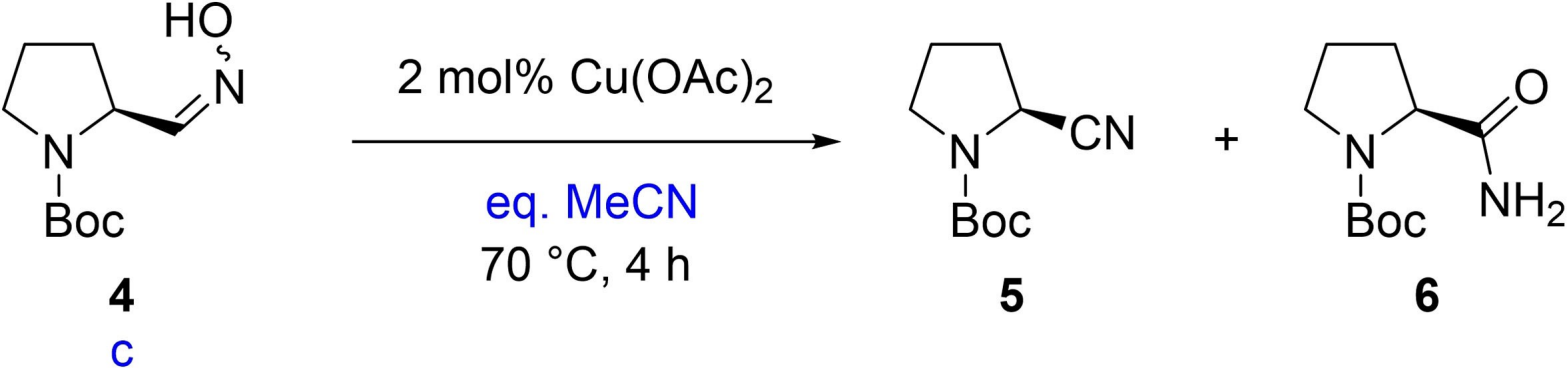					
Entry	Equiv. MeCN	Substrate concentration [mm]	Aldoxime **4** [%]	Nitrile **5** [%]	Amide **6** [%]
**1**	235	80	–	99	1
**2**	150	127	–	96	4
**3**	125	152	–	96	4
**4**	100	190	–	95	5
**5**	75	253	–	94	6
**6**	64	300	–	93	7
**7**	48	400	–	91	9
**8**	38	500	–	89	11
**9**	10^[a]^	500	–	74	26
**10**	10^[a]^	300	–	73	27
**11**	10^[a]^	100	9	68	23

[a] Ethyl acetate was used as solvent.

In contrast, a reduction of the substrate concentration to 100 mm using 10 equivalents of acetonitrile in ethyl acetate as a solvent only leads to a reduced reaction rate, but not to a relevant reduction of the formed undesired proline amide **6** in the reaction mixture (entry 11).

These results also support the described mechanism of an amide formation via a nitrile intermediate. Accordingly, an increased amount of acetonitrile as acceptor nitrile and, thus, co‐substrate decreases the probability of a further reaction of the desired Boc‐protected nitrile **5** as such a co‐substrate in the further course of the reaction.

**Scheme 3:**
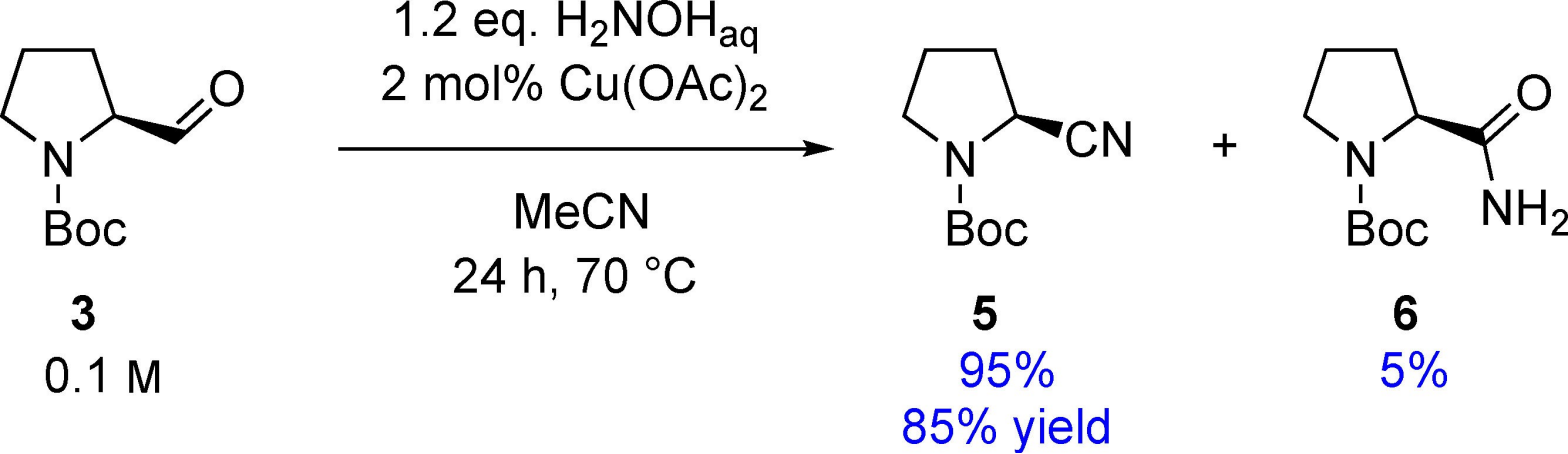
One‐pot synthesis of *N*‐Boc‐(S)‐2‐cyanopyrrolidine (**5**) in pure acetonitrile.

With these findings on the impact of various reaction conditions on the dehydration of aldoxime **4** in hand, we next focused on the preparation of Boc‐2 cyanopyrrolidine **5** within a one‐pot process starting from aldehyde **3** (Scheme 3). The reaction was carried out at a substrate concentration of 0.1 m using an aqueous hydroxylamine solution in acetonitrile.

Compared to the one‐step dehydration of prolinal oxime **4**, a longer reaction time was needed to achieve full conversion of the starting material. After 24 h, *N*‐Boc‐(*S*)‐2‐cyanopyrrolidine (**5**) was isolated in a yield of 85 %, whereas only 5 % conversion to amide **6** was observed (Scheme 3). Note that an optical rotation analysis revealed a full retention of the chiral information.

Although using a large excess of acetonitrile enables suppression of the side product and high conversion, the resulting substrate concentrations of 80 or 100 mm are not attractive for an industrial application. Thus, other options to inhibit amide formation have been considered and, in particular, we became interested in a fine‐tuning of the electronic properties of the acceptor nitrile with respect to the ability to add water within the dehydration step with aldoxime **4** as a substrate. We envisioned that reducing the electron density at the carbon atom of the nitrile moiety of the “acceptor nitrile” by electron‐withdrawing substituents could lead to a faster (formal) addition of water during the dehydration process of the aldoxime substrate. In addition, the electronic properties of the aldoxime substrate should then play a role on both conversion and side product formation. While a general influence of electron donating substituents in this type of reaction is mentioned, an influence on amide formation under the chosen conditions has not yet been described.^[24]^

**Figure 2:**
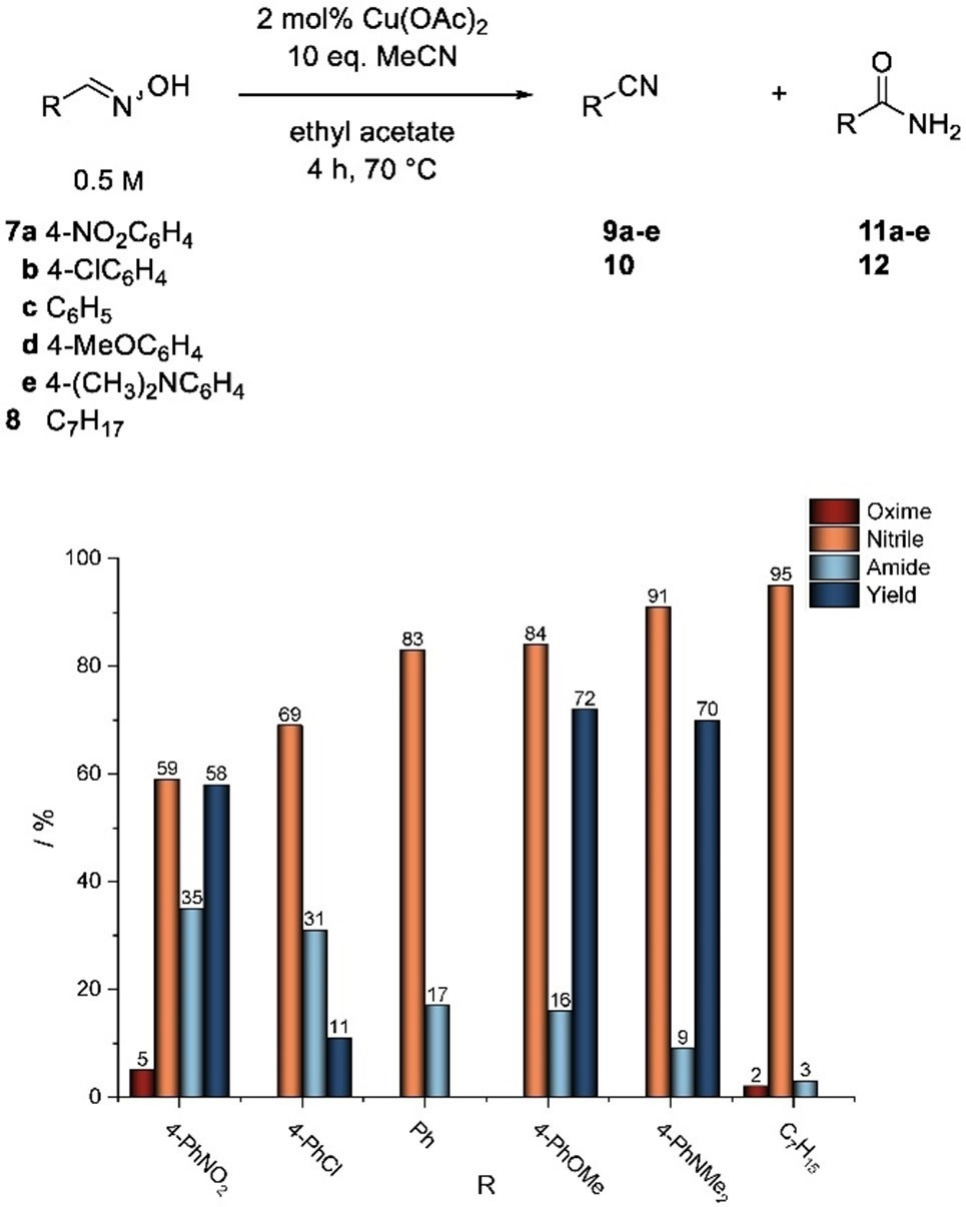
Influence of substrate structure on dehydration of aldoximes using acetonitrile as an acceptor nitrile.

In order to verify this hypothesis, at first the influence of the electron density at the aldoxime on amide formation in the standard reaction was investigated (Figure 2). For this purpose, besides *n*‐octanal oxime, various *p*‐substituted benzaldoximes were chosen based on their Hammett values and used as substrates in a dehydration reaction.

Initially, the benzaldoximes **7 a**–**e** with different substitution pattern in 4‐position were prepared from the respective aldehydes in good to excellent yields. Similarly, amides **11 a**‐**e** were obtained from the corresponding aldoximes as reference compounds in low to moderate yields by a copper(II)‐catalyzed rearrangement in toluene. The Hammett values σ_p_ of the aromatic aldoximes ranged from 0.78 for *p‐*nitrobenzaldoxime **7 a** to −0.83 for *p*‐(*N*,*N*‐dimethyl)aminobenzaldoxime **7 e**, defining the electronic characteristics of the *p*‐substituents. Furthermore, *n*‐octanal oxime (**8**) was investigated as an aliphatic substrate. We were pleased to find that the dehydration reaction under standard conditions (Scheme 1) indeed proceeded for all selected substrates. However, there is a considerable difference in the selectivity of the reaction.

For the aliphatic substrate *n*‐octanal oxime (**8**), only a small amount of the side product *n*‐octanamide (**12**) (3 %) is observed at complete conversion of the aldoxime (Figure 2).

When using the 4‐substituted benzaldoximes **7 a**–**e** as substrates, there is a clear dependency of the selectivity on the Hammett parameter and, thus, on the electron density at the carbon atom of the aldoxime group (Figure 2). When using benzaldoxime **7 e** containing the dimethylamino group as the most electron‐donating substituent in this study as a substrate, formation of the resulting benzamide **11 e** in only 9 % yield was observed. However, when reducing the degree of electron donation, associated with an increasing σ_p_ value, the selectivity decreases sharply. While in the dehydration of unsubstituted benzaldoxime **7 c** to benzonitrile **9 c** already 17 % amide is formed, the selectivity decreases further for electron‐withdrawing substituents. An amide formation of 31 % and 35 % in the dehydrations towards *p*‐chlorobenzonitrile **9 b** (σ_p_=0.23) and *p*‐nitrobenzonitrile **9 a** (σ_p_=0.78), respectively, was observed, although only a conversion of 95 % was noted in the latter case. These observations confirm that the reactivity of the aldoxime and the resulting nitrile significantly influence the progress and selectivity of the reaction. The electron‐withdrawing substituent in *para‐*position lowers the electron density at the cyanocarbon of the in situ formed *p*‐nitrobenzonitrile **9 a**, favoring a nucleophilic attack in the dehydration of unreacted aldoxime **7 a** compared to the higher electron density of acetonitrile, which served as the acceptor nitrile in the initial dehydration step. In the conversion of octanal oxime **8**, this effect is only slightly observed, since the resulting octanenitrile **10** has a similar electron density at the reactive center as acetonitrile. Here, the 10‐fold excess of acetonitrile plays a more significant role, which leads to the suppression of the undesired amide formation in this case.

### 2.3 Variation of Acceptor Nitrile

**Figure 3:**
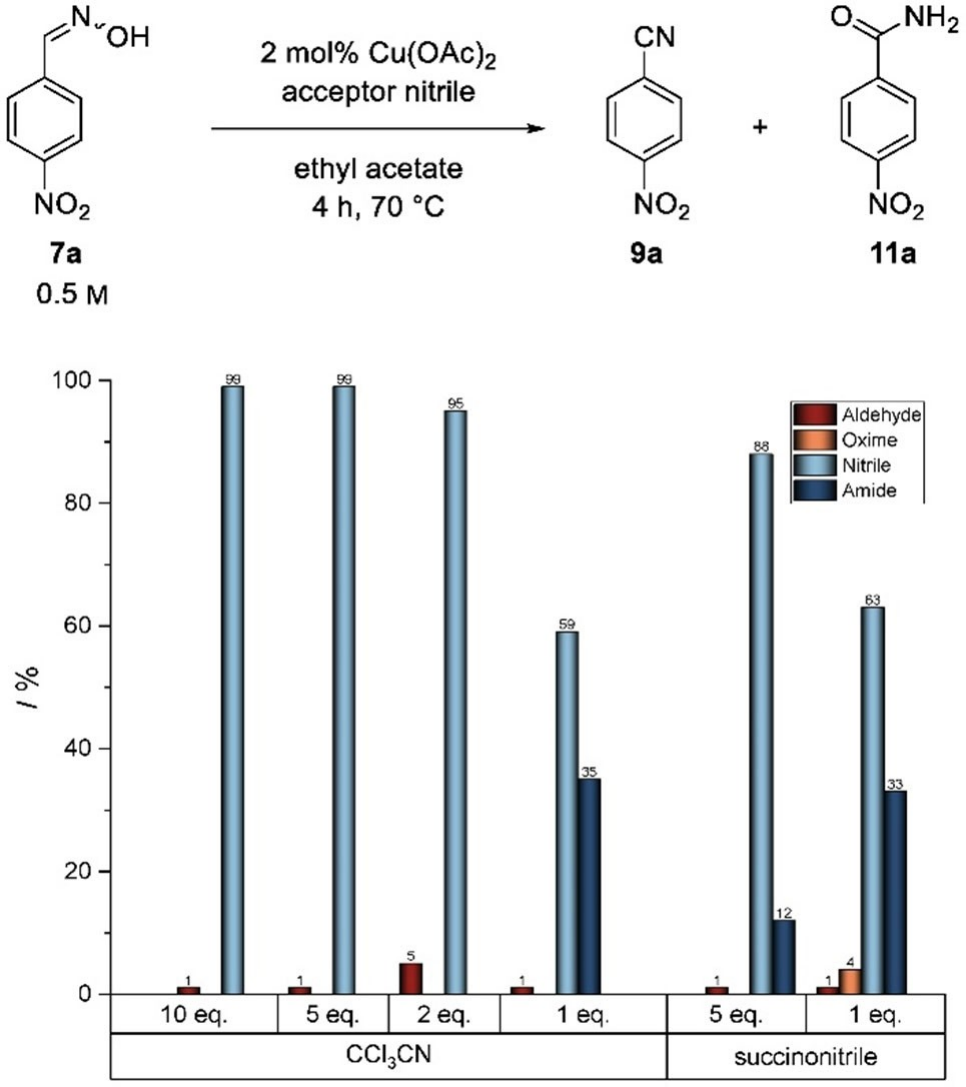
Influence of different acceptor nitriles on dehydration of *p*‐nitro benzaldoxime **7 a**.

Based on these findings, a modification of the acceptor nitrile with regard to suppression of amide formation was investigated next. At first, different monosubstituted, aliphatic acceptor nitriles such as butyronitrile or octanenitrile were used, but these acceptor nitriles showed no positive influence on the selectivity in the dehydration of prolinal oxime **4** (see Supporting Information). However, when using more electrophilic acceptor nitriles we found a significant increase of the reactivity with respect to water addition, as expected. In detail, we tested two different acceptor nitriles with such an expected higher reactivity, namely trichloroacetonitrile and succinonitrile (Figure 3). Trichloroacetonitrile exhibits a strong electrophilicity due to the trifold chlorine substitution, whereas succinonitrile has just a slightly lower electron density at the cyano‐carbon atom compared to acetonitrile, but has the advantage of a bifunctionality. This bifunctionality theoretically enables to halve the number of equivalents compared to those needed for acetonitrile.

The reaction was first carried out for both acceptor nitriles under standard conditions with *p*‐nitrobenzaldoxime **7 a** as aldoxime substrate, which, in previous experiments, had shown the highest tendency to amide formation. Ten equivalents of the nitrile functionalities were used in each case in the first place, resulting in the conversion of aldoxime **7 a** to nitrile **9 a** in both cases. When five molar equivalents of succinonitrile are used, a significant improvement of the selectivity (12 % amide) was observed in comparison with acetonitrile (35 % amide). On the other hand, reduction to two molar equivalents of succinonitrile leads to a non‐complete conversion (96 %) of **7 a** after 4 h with a similarly high amide **11 a** content (33 %) compared to the experiment with ten equivalents of acetonitrile. When using trichloroacetonitrile as a co‐substrate, we were pleased to find that this compound showed even better properties as a nitrile acceptor (Figure 3). Here, a complete conversion of the *para*‐substituted benzaldoxime **7 a** to nitrile **9 a** was observed in the presence of 10, 5 and 2 equivalents. Furthermore, extractive and column chromatographic work‐up gave the resulting product 4‐nitrobenzonitrile **9 a** in 89 % yield, which is a considerable increase compared to the synthesis using acetonitrile (58 %). Only with just an equimolar use of this acceptor nitrile, analogous to succinonitrile, an incomplete conversion and, with 35 %, a high proportion of the undesired *p*‐nitrobenzamide **11 a** as a side product was observed.

**Scheme 4:**
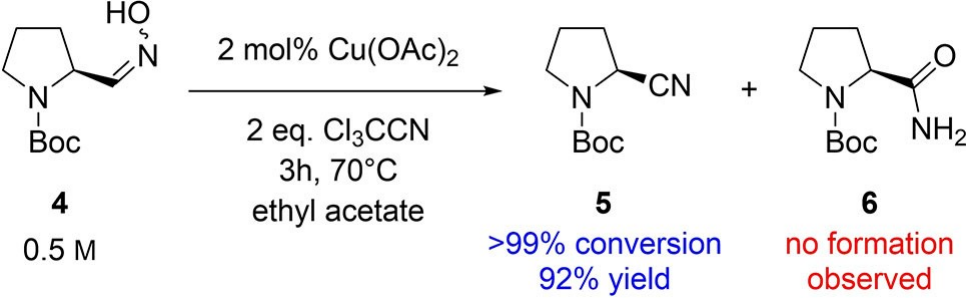
Dehydration of Boc‐prolinal oxime **4** with trichloroacetonitrile as the optimized nitrile acceptor.

With this improved nitrile acceptor in hand, these findings were applied to the dehydration of Boc‐prolinal oxime **4** as a next step. When utilizing either ten equivalents (data not shown) or even just two equivalents of trichloroacetonitrile, we were pleased to find a complete conversion of the aldoxime without formation of the undesired amide side‐product (Scheme 4). For the experiment with two equivalents of trichloroacetonitrile, work‐up and product isolation were conducted, which led to the isolation of the desired *N*‐Boc‐(*S*)‐2‐cyanopyrrolidine **5** in 92 % yield. Furthermore, also in this case full retention of the absolute configuration was observed.

## 3 Conclusion

In this work, the relationship between the ratio of the reactivity of the acceptor and product nitrile and its influence on the formation of the undesired amide side product in a dehydration of aldoximes was investigated. Toward this end, the dehydration of *para*‐substituted benzaldoximes with different electron densities at the carbon atoms of the aldoxime and cyano groups and using acetonitrile as the co‐substrate was analyzed. Depending on the chosen substrate, amide formation can be reduced or completely suppressed by a high excess of acetonitrile or by appropriate choice of a more electrophilic acceptor nitrile such as trichloroacetonitrile. By suppressing the undesired amide formation, the yields were significantly improved for *p‐*nitrobenzonitrile **9 a** and *N*‐Boc‐cyanopyrrolidine **5**, leading to a high yield of 92 % in the latter case.

## Conflict of interest

The authors declare no conflict of interest.

4

## Supporting information

As a service to our authors and readers, this journal provides supporting information supplied by the authors. Such materials are peer reviewed and may be re‐organized for online delivery, but are not copy‐edited or typeset. Technical support issues arising from supporting information (other than missing files) should be addressed to the authors.

Supporting Information

## Data Availability

The data that support the findings of this study are available from the corresponding author upon reasonable request.
