# Supplementary material for: Process Development of the Copper(II)‐Catalyzed Dehydration of a Chiral Aldoxime and Rational Selection of the Co‐Substrate
Source: ChemistryOpen. 2021 Dec 10;11(1):e202100230. doi: 10.1002/open.202100230 (PMC8734112; doi:10.1002/open.202100230)
Supplement: Supplementary file 1 — Supporting Information [file OPEN-11-e202100230-s001.pdf]

ChemistryOpen

Supporting Information

Process Development of the Copper(II)-Catalyzed Dehydration of a Chiral Aldoxime and Rational Selection of the Co-Substrate

Jannis Nonnhoff and Harald Gröger*

Table of contents

1. Analytical Data 2

2. Experimental Procedures 4

Introduction experiment 4

Screening of reaction conditions for dehydration of aldoximes 5

General Procedure 1 (GP1): Synthesis of benzaldoximes 8

General Procedure 2 (GP2): Synthesis of amides 10

Investigation of copper-catalyzed dehydration of 4-substitued benzaldoximes 12

One-pot process with 0.1 M substrate loading in acetonitrile 14

3. Spectroscopic Data 15

4. References 17

1. Analytical Data

Chemical were purchased from TCI Chemicals, Fluorochem, Sigma Aldrich and VWR Chemicals and were used without further purification.

NMR-spectra were recorded on a Bruker Avance III at a frequency of 500 MHz (^{1}H) or 126 MHz (^{13}C). The chemical shift δ is given in ppm and referenced to the solvent signal of deuterized Chloroform. Coupling constants (*J*) were given in hertz.

Nano-ESI mass spectra were recorded using an Esquire 3000 ion trap mass spectrometer from Bruker Daltonik GmbH equipped with a nano-ESI source. Samples were induced by static nano-ESI using inhouse pulled glass emitters. Nitrogen served as nebulizer and dry gas. Helium was used as cooling gas for the ion trap. The mass axis was externally calibrated with the ESI-L Tuning Mix from Agilent Technologies. The spectra were recorded by accumulation and averaging of every single spectra using Bruker Daltonik esquireNT 5.2. esquireControl software. For processing of the spectra, DataAnalysis software 3.4 was used.

Conversions were determined by GC measurements using a GC-2010 Plus from Shimadzu in comparison to GC-areas. Following methods were used.

Method 1: chiral SGE Analytik B&B-174 column, 160 °C initial temperature, 2 °C/min temperature ramp, 1 min 180 °C, -50 °C/min temperature ramp, end temperature 50 °C.

Method 2: *Zebron ZB-Fame*, 130 °C initial temperature, 10 °C/min temperature ramp, 4 min 240 °C.

Method 3: *Zebron ZB-Fame*, 130 °C initial temperature, 40 °C/min temperature ramp, 4 min 240 °C, R_t (Nitrile) = 2.19 min, R_t (Oxime) = 2.71 min, R_t (Amide) = 5.04 min.

In Table 1, retention times of all products and by-products are listed.

Table 1: Retention time of *N*-Boc-L-proline derivatives.

**Method**	**Compound**	**Retention time / min**
1	*N*-Boc-L-prolinal	4.8
	N-Boc-(*S*)-2-cyanopyrrolidine	6.9
	N-Boc-L-prolinal oxime	11.8
	N-Boc-L-prolinamide	15.2
	N-Boc-L-proline	18.1
2	Octanenitrile	3.2
	Octanaloxime	5.9
	Octanamide	10.9
3	Benzonitrile	2.1
	Benzaldoxime	2.7
	Benzamide	5.0

2. Experimental Procedures

Introduction experiment

Synthesis of *N*-boc-L-prolinal oxime

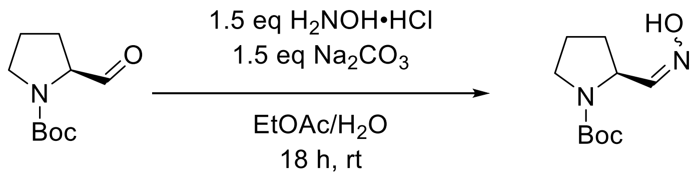

0.5 M

A solution of hydroxylamine hydrochloride (1.5 eq., 15.7 g, 226 mmol) and sodium bicarbonate (0.75 eq. 12.0 g, 113 mmol) in water (150 mL) was added to a solution of *N*-Boc-L-prolinal (1.0 eq., 30.0 g, 151 mmol) in ethyl acetate (150 mL) and stirred for 8 h at room temperature. The phases were separated and the aqueous phase extracted three times with ethyl acetate (each 150 mL). Combined organic layers were dried over magnesium sulfate and evaporated in vacuo. *N*-Boc-L-prolinal oxime (30.6 g, 142.4 mmol, 95%, *E*/*Z* ratio 70:30) was obtained as a colorless oil.

^{1}H-NMR (500 MHz, $CDCl_3$): δ = 8.49 (s, 0.1H, CNO*H*), 8.32 (s, 0.2H, CNO*H*), 8.14 (s, 0.4H, CNO*H*), 8.07 (s, 0.3H, CNO*H*), 7.40+7.31 (2s, 0.7H, C*H*NOH), 6.74+6.68 (2s, 0.3H, C*H*NOH), 4.89+4.83 (2s, 0.3H, C*H*CHNOH), 4.46+4.32 (2s, 0.7H, C*H*CHNOH), 3.53–3.28 (m, 2H, NCH_2), 2.32–1.79 (m, 4H, $NCH_2CH_2CH_2$), 1.44 (s, 9H, 3 CH_3).

^{13}C-NMR (126 MHz, $CDCl_3$): δ = 154.5+154.3 (N*C*(O)O), 151.4+151.0 (*C*NOH), 80.1+79.9 (*C*(CH_3)), 56.2+55.9+52.8 (*C*HCHNOH), 46.7+46.6+46.4 (NCH_2), 30.7+30.0+29.4 ($NCH_2CH_2CH_2$), 28.5+28.4 (3 CH_3), 24.9+24.3+24.0+23.4 ($NCH_2CH_2CH_2$).

MS (ESI): *m*/z (%) = 237.0 (7), 451.2 (100).

Synthesis of *N*-boc-(*S*)-2-cyanopyrrolidin

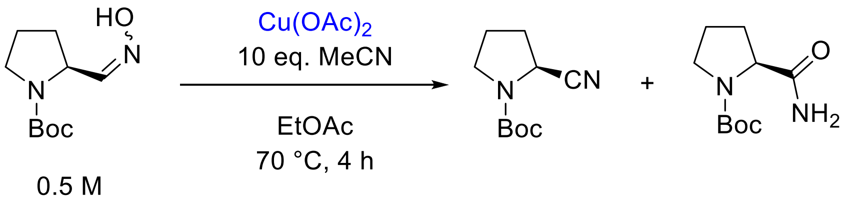

0.5 M

Copper(II) acetate (2 mol%, 0.8 mg, 4.6 µmol) and acetonitrile (10 eq., 94 mg, 2.30 mmol) were added to a solution of *N*-Boc-L-prolinal oxime (1.0 eq., 50 mg, 0.23 mmol) in ethyl acetate (0.34 mL). The mixture was stirred for 18 h at 70 °C. Full conversion of the oxime was

determined via GC. The solvent was evaporated and the green crude product purified by filtration over silica (cyclohexane/ethyl acetate 2:1). After evaporation of the solvent, *N*-Boc-2-(*S*)-cyanopyrrolidine was obtained as a colorless oil (36 mg, 0.19 mmol, 80%).

^{1}H-NMR (500 MHz, $CDCl_3$): δ = 4.61–4.50 (m, 0.4 H, CHCN), 4.50–4.41 (m, 0.6 H, C*H*CN), 3.58–3.43 (m, 1H, $NCH_2CH_2CH_2$), 3.42–3.26 (m, 1H, $NCH_2CH_2CH_2$), 2.33–1.93 (m, 4H, $NCH_2CH_2CH_2$), 1.51+1.48 (2 s, 9H, 3 CH_3).

^{13}C-NMR (126 MHz, $CDCl_3$): δ = 153.8+153.1 (N*C*(O)O), 119.2 (CN), 81.5+81.1 (*C*(CH_3)), 47.3+47.1 (*C*HCN), 46.1+45.8 (N*C*H_2), 31.8+30.9 ($NCH_2CH_2CH_2$), 28.6 + 28.4 (3 CH_3), 24.8+23.9 ($NCH_2CH_2CH_2$).

MS (ESI): *m*/*z* (%) = 219.0 (37), 415.6 (100).

Screening of reaction conditions for dehydration of aldoximes

<u>Dehydration of *N*-boc-L-prolinal oxime: catalyst screening</u>

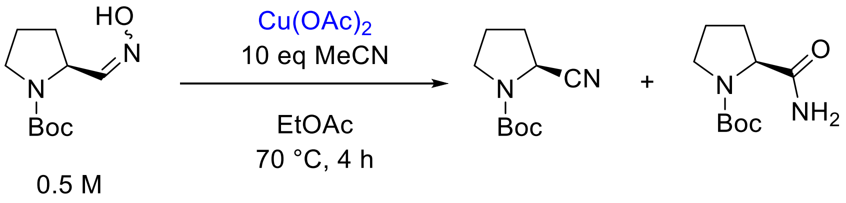

Copper(II) acetate (4.65 to 46.5 µmol) was added as 1 M solution in acetonitrile (total amount in each reaction 489 µL, 10 eq.) to a solution of *N*-boc-L-prolinal oxime (1.00 eq., 0.20 g, 0.93 mmol) and ethyl acetate (1.37 mL). The solution was stirred for 4 h at 70 °C. An aliquot was taken and analyzed via GC. Conversions are mentioned in Table 2.

Table 2: Composition of the reaction solutions after 4 h with respect to catalyst loading.

$Cu(OAc)_2$ [mol%]	oxime [%]	nitrile [%]	amide [%]
**0.5**	62	28	10
**1**	0	79	21
**2**	0	82	18
**5**	0	82	18

Dehydration of *N*-boc-L-prolinal oxime: temperature screening

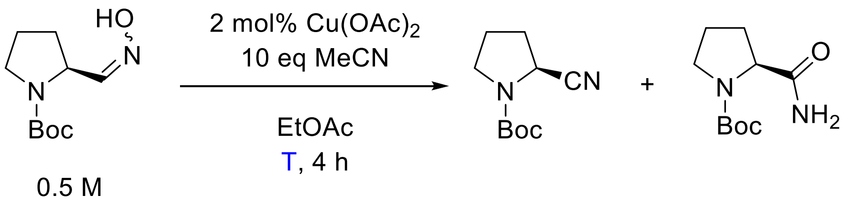

Copper(II) acetate (2 mol%, 0.34 mg, 2.0 µmol) was added to a solution of *N*-Boc-L-prolinal oxime (1.0 eq. 21.4 mg, 0.10 mmol) in acetonitrile (10 eq., 40.8 mg, 1.0 mmol, 52.3 µL) and ethyl acetate (147.8 µL). The solution was heated to 70 °C and stirred for 4 h. An aliquot (10 µL) was taken and filtrated over silica with ethyl acetate (1 mL) to determine the conversion via GC.

Table 3: Conversions of Boc-prolinal oxime at different temperatures

**T / °C**	**Time / h**	**Oxime / %**	**Nitrile / %**	**Amide / %**
70	4	-	71	29
50	4	-	67	33
	18	-	63	37
30	4	7	64	30
	18	-	63	37
rt	4	21	63	16
	18	-	68	32
rt[a]	18	2	65	32
rt[b]	18	3	67	30
-20[a]	18	81	14	5
rt[c]	18	97	2	1
70[c]	18	80	7	13

A: without stirring, b: 300 mg batch, c: without acetonitrile.

Influence of equivalents of acetonitrile on amide formation

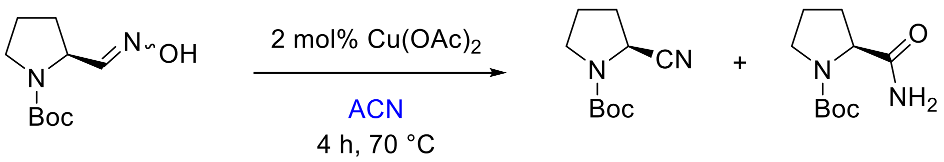

Copper(II) acetate (2 mol%, 1.7 mg, 9.4 µmol) was added to a solution of *N*-Boc-L-prolinal oxime (1.0 eq. 100 mg, 0.47 mmol) in acetonitrile (0.94 – 3.71 mL). The solution was heated to 70 °C and stirred for 4 h. An aliquot (10 to 100 µL) was taken and filtrated over silica with

ethyl acetate (1 mL) to determine the conversion via GC. Composition of the reaction solutions are shown in Table 4.

Screening of reaction conditions: substrate loading 100-500 mM

Copper(II) acetate (0.02 eq., 2.5 mg, 14 µmol) and acetonitrile (10.0 eq. 287 mg, 7.0 mmol) were added to a solution of *N*-Boc-L-prolinal oxime (1.0 eq. 150 mg, 0.7 mmol) in ethyl acetate or acetonitrile (1.032 mL, 1.962 mL, 6.632 mL). The solution was stirred at 70 °C for 4 h. Aliquots (10 µL, 20 µL, 50 µL) were taken and filtrated over silica with ethyl acetate (1 mL) to determine conversion via GC. Composition of the reaction solutions are shown in Table 4.

Table 4: Composition of the reaction solutions after 4 h with respect to substrate loading and equivalents of acetonitrile.

**ACN / Eq.**	**Substrate loading / mM**	**Aldehyde / %**	**Oxime / %**	**Nitrile / %**	**Amide / %**
235	80	-	-	99	1
150	127	-	-	96	4
125	152	-	-	96	4
100	190	-	-	95	5
75	253	-	-	94	6
64	300	-	-	93	7
48[a]	400	-	-	91	9
38[a]	500	-	-	89	11
10[b]	500	3		72	25
10[b]	300	3		71	26
10[b]	100	4	9	65	22

[a] batch size was doubled; [b] ethyl acetate was used as solvent.

Substrate Influence

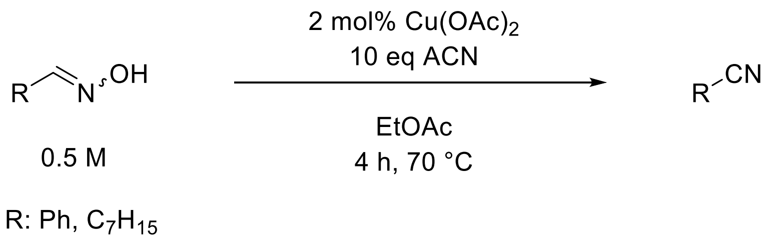

Copper(II) acetate (2 mol%) and acetonitrile (10 eq.) were added to a solution of (*E*)-benzaldoxime or *n*-octanal oxime (1.0 eq.) in ethyl acetate (total substrate concentration 0.5 M). The solution was heated to 70 °C and stirred for 4 h. An aliquot (80 µL) was taken and filtrated over silica with ethyl acetate (1 mL) to determine the conversion via GC.

Table 5: Composition of the reaction solutions after 4 h with respect to the substrate.

Substrate	Oxime / %	Nitrile / %	Amide / %
(*E*)-Benzaldoxime	-	83	17
n-Octanal oxime	2	95	3

General Procedure 1 (GP1): Synthesis of benzaldoximes

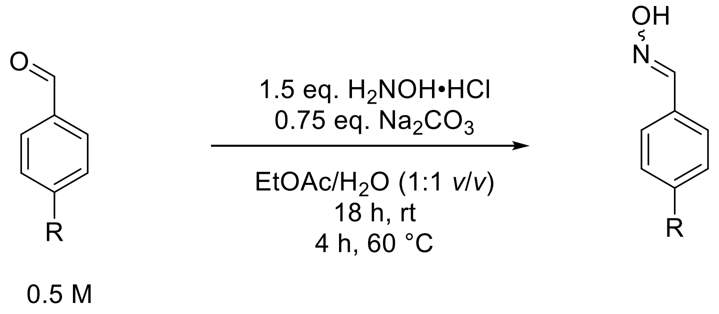

An aqueous solution of hydroxylamine hydrochloride (1.5 eq.) and sodium carbonate (0.75 eq.) was added to a solution of 4-substituated benzaldehyde (1.0 eq.) in ethyl acetate (total concentration 0.5 M, 1:1 *v*/*v* water/ethyl acetate). The solution was stirred at room temperature for 18 h and afterwards heated to 60 °C and stirred for further 4 h. The phases were separated und the aqueous layer extracted three times with ethyl acetate (1:1 *v*/*v*). After drying over magnesium sulfate and evaporation of the solvent, the benzaldoximes are obtained.

Benzaldoxime

The synthesis was done following GP1 using undistilled benzaldehyde (1.00 g, 9.42 mmol), hydroxylamine hydrochloride (2.0 eq., 1.31 g, 18.8 mmol), sodium carbonate (1.0 eq., 0.998 g, 9.42 mmol), water (9.42 mL) and ethyl acetate (9.42 mL). The crude product was purified by column chromatography (5-40% ethyl acetate in cyclohexane). The different isomers could be separated. (*E*)-Benzaldoxime was obtained as a colorless oil (0.681 g, 5.62 mmol, 60%), (*Z*)-benzaldoxime as a colorless solid (0.05 g, 0.40 mmol, 4%). The ^{1}H-NMR data correspond to the literature.[1]

(*E*)-Benzaldoxime:

^{1}H-NMR (500 MHz, $CDCl_3$): δ [ppm] = 8.15 (s, 1H), 7.74 (s, 1H), 7.61 – 7.55 (m, 2H), 7.41 – 7.37 (m, 3H).

(*Z*)-Benzaldoxime:

^{1}H-NMR (500 MHz, $CDCl_3$): δ [ppm] = 7.94 (dd, J = 7.6, 2.2 Hz, 2H), 7.47 – 7.35 (m, 5H).

4-Chlorobenzaldoxime

The synthesis was done following GP1 using 4-chlorobenzaldehyde (1.00 g, 7.11 mmol), hydroxylamine hydrochloride (0.741 g, 10.7 mmol), sodium carbonate (0.564 g, 5.33 mmol), ethyl acetate (7 mL) and water (7 mL). 4-Chlorobenzaldoxime could be obtained as a colorless solid (0.924 g, 5.94 mmol, 84%) with an *E*/*Z*-ratio of approximately 1:11. The ^{1}H-NMR spectroscopic data are in accordance with the literature.[2]

^{1}H NMR (500 MHz, $CDCl_3$) *δ* [ppm] = 8.20 – 8.13 (m, 1H, NO*H*), 8.11 (s, 1H C*H*NOH), 7.92 – 7.88 (m, 0.2H, *E*-C*H*CCHNOH)), 7.53 – 7.49 (m, 2H, *Z*-C*H*CCHNOH), 7.43 – 7.39 (m, 0.2H, *E*-C*H*CCl), 7.39 – 7.33 (m, 2H, *Z*-C*H*CCl).

ESI-MS: *m/z* [Fragment] = 154.8 [M-H].

4-Methoxybenzaldoxime

The synthesis was done following GP1 using 4-methoxybenzaldehyde (1.00 g, 7.34 mmol), hydroxylamine hydrochloride (0.765 g, 11.0 mmol), sodium carbonate (0.584 g, 5.51 mmol), ethyl acetate (7.34 mL) and water (7.34 mL). 4-Methoxybenzaldoxime could be obtained as a colorless solid (1.08 g, 7.14 mmol, 97%) with an *E*/*Z*-ratio of approximately 1:11. The ^{1}H-NMR spectroscopic data are in accordance with the literature.[2]

^{1}H NMR (500 MHz, $CDCl_3$) *δ* [ppm] = 8.10 (s, 1H, *Z*-C*H*NOH), 8.01 – 7.91 (bs 1H, NO*H* + m, 0.2H, *E*-C*H*CCHNOH), 7.55 – 7.49 (m, 2H, *Z*-C*H*CCHNOH), 7.31 (s, 0.1H, *E*-C*H*NOH), 6.96 – 6.93 (m, 0.2H, *E*-C*H*COMe), 6.93 – 6.88 (m, 2H, *Z*-C*H*$COCH_3$), 3.85 (s, 0.3H, OCH_3), 3.83 (s, 3H, OCH_3).

ESI-MS: *m/z* [Fragment] = 151.9 [M+H], 173.9 [M+Na], 353.3 [2M+H], 381.3 (2M+Na).

4-Nitrobenzaldoxime

The synthesis was done following GP1 using 4-nitrobenzaldehyde (1.00 g, 6.62 mmol), hydroxylamine hydrochloride (0.690 g, 9.93 mmol), sodium carbonate (0.526 g, 4.97 mmol), ethyl acetate (6.6 mL) and water (6.6 mL). 4-Nitrobenzaldoxime could be obtained as a yellow solid (1.04 g, 6.23 mmol, 94%) an *E*/*Z*-ratio of approximately 1:11. The ^{1}H-NMR spectroscopic data is in accordance with the literature.[2]

^{1}H NMR (500 MHz, $CDCl_3$) *δ* [ppm] = 8.30 – 8.27 (m, 0.2H, *E*-C*H*CNO_2), 8.26 – 8.22 (m, 2H, *Z*-C*H*CNO_2), 8.20 (s, 1H, *Z*-C*H*NOH), 8.12 – 8.09 (M, 0.2H, *E*-C*H*CCHNOH), 8.01 (bs, 1H, NO*H*), 7.77 – 7.72 (m, 2H, *Z*-C*H*CCHNOH), 7.51 (s, 0.1H, *E*-C*H*NOH).

ESI-MS: *m/z* [Fragment] = 200.8 [M-H].

4-(*N*,*N*-Dimethylamino)benzaldoxime

The synthesis was done following GP1 using 4-(*N*,*N*-dimethylamino)benzaldehyde (1.00 g, 6.70 mmol), hydroxylamine hydrochloride (0.698 g, 10.1 mmol), sodium carbonate (0.533 g, 5.03 mmol), ethyl acetate (6.7 mL) and water (6.7 mL). 4-(*N*,*N*-Dimethylamino)benzaldoxime could be obtained as a colorless solid (0.923 g, 5.66 mmol, 84%) with an *E*/*Z*-ratio of approximately 1:11. The ^{1}H-NMR spectroscopic data are in accordance with the literature.[3]

^{1}H NMR (500 MHz, $CDCl_3$) δ [ppm] = 8.06 (s, 1H, *Z*-C*H*NOH + bs, 1H, NO*H*), 7.90 – 7.84 (m, 0.2H, *E*-C*H*CCHNOH), 7.47 – 7.40 (m, 2H, *Z*-C*H*CCHNOH), 7.24 (s, 0.1H, *E*-C*H*NOH), 6.72 – 6.64 (m, 2H, *Z*-C*H*CCN(CH_3)$_2$ + m, 0.2, *E*-C*H*CCN(CH_3)$_2$), 3.02 (s, 0.5H), 3.00 (s, 6H).

ESI-MS: *m/z* [Fragment] = 165.0 [M+H], 187.0 [M+Na].

General Procedure 2 (GP2): Synthesis of amides

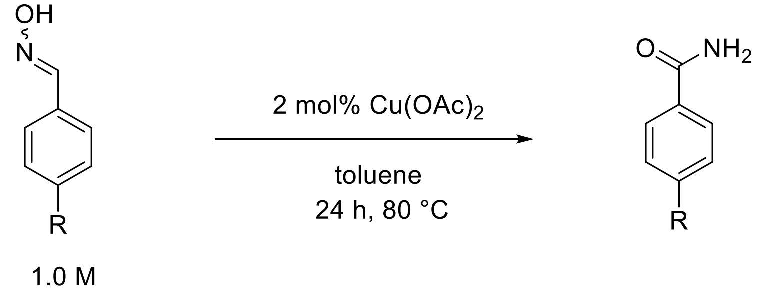

The synthesis was done according to Sharma *et al*.[4] Copper(II) acetate (2 mol%) was added to a solution or suspension of 4-substituated benzaldoximes (1.0 eq.) in toluene (1 M). The reaction was heated to 80 °C for 24 h. The solvent was removed, the crude product resuspended in dichloromethane and afterwards purified by column chromatography (50–100% ethyl acetate in cyclohexane). The 4-substituated benzamides could be obtained after evaporation of the solvent.

4-Chlorobenzamide

The synthesis was done according to GP2 using copper(II) acetate (2.3 mg, 0.01 mmol), 4-chlorobenzaldoxime (100 mg, 0.64 mmol) and toluene (0.64 mL). 4-Chlorobenzamide could be obtained as a colorless solid (64 mg. 0.41 mmol, 68%). The ^{1}H-NMR spectroscopic data are in accordance with the literature.[5]

^{1}H NMR (500 MHz, $CDCl_3$) δ [ppm] = 7.78 – 7.72 (m, 2H, C*H*CCONH$_2$), 7.48 – 7.38 (m, 2H, C*H*CCl), 5.94 (bd, J = 79.9 Hz, 2H, CON*H*$_2$).

ESI-MS: *m/z* [fragment] = 154.0 [M-H].

4-Methoxybenzamide

The synthesis was done according to GP2 using copper(II) acetate (3.6 mg, 0.02 mmol), 4-methoxybenzaldoxime (150 mg, 0.99 mmol) and toluene (0.99 mL). 4-Methoxybenzamide could be obtained as a colorless solid (34 mg. 0.22 mmol, 23%). The ^{1}H-NMR spectroscopic data are in accordance with the literature.[5]

^{1}H NMR (500 MHz, $CDCl_3$) *δ* [ppm] = 7.82 – 7.75 (m, 2H), 6.97 – 6.89 (m, 2H), 5.91 (s, 1H), 5.62 (s, 1H), 3.86 (s, 3H).

ESI-MS: *m/z* [fragment] = 151.9 [M+H].

4-Nitrobenzamide

The synthesis was done according to GP2 using copper(II) acetate (1.8 mg, 0.01 mmol), 4-chlorobenzaldoxime (100 mg, 0.60 mmol) and toluene (0.60 mL). 4-Chlorobenzamide could be obtained as a yellow solid, contaminated with 40% oxime (43 mg, leading to 0.16 mmol, 26%). The ^{1}H-NMR spectroscopic data are in accordance with the literature.[5]

^{1}H NMR (500 MHz, $CDCl_3$) *δ* [ppm] = 8.34 – 8.30 (m, 2H, $CHCNO_2$), 8.01 – 7.97 (m, 2H, $CHCCONH_2$), 6.10 (bs, 1H, $CONH_2$), 5.78 (bs, 1H, $CONH_2$).

ESI-MS: *m/z* [fragment] = 164.8 [M-H].

4-(*N*,*N*-Dimethylamino)benzamide

The synthesis was done according to GP2 using copper(II) acetate (1.8 mg, 0.01 mmol), 4-(*N*,*N*-dimethylamino)benzaldoxime (100 mg, 0.61 mmol) and toluene (0.61 mL). 4-(*N*,*N*-Dimethylamino)benzamide could be obtained as a colorless solid (17 mg. 0.1 mmol, 16%). The ^{1}H-NMR spectroscopic data are in accordance with the literature.[5]

^{1}H NMR (500 MHz, $CDCl_3$) *δ* [ppm] = 7.74 – 7.69 (m, 2H, $CHCCONH_2$), 6.73 – 6.62 (m, 2H, $CHCN(CH_3)_2$), 5.75 (bs, 2H, $CONH_2$), 3.05 + 3.03 (2s, 6H, $N(CH_3)_2$).

ESI-MS: *m/z* [fragment] = 187.0 [M+Na].

Investigation of copper-catalyzed dehydration of 4-substitued benzaldoximes

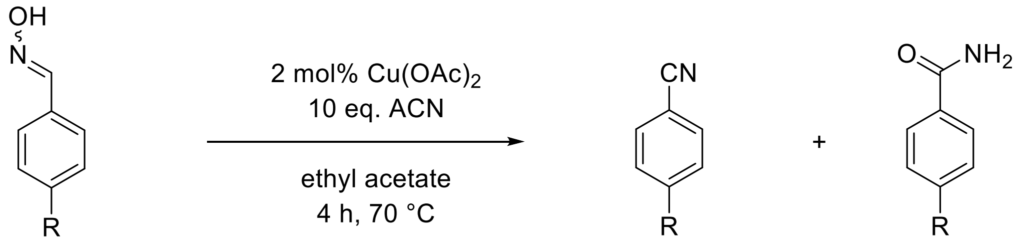

0.5 M

R = H
= Cl
= OMe
= NO_2
= NMe_2

Copper(II) acetate (2 mol%) and acetonitrile (10 eq.) were added to a solution of 4-substituted benzaldoxime (1.0 eq., 150 mg) in ethyl acetate (total substrate loading of 0.5 M). The solution was heated to 70 °C and stirred for 4 h. An aliquot (50 µL) was taken and analyzed via ^{1}H-NMR spectroscopy. The conversions and σ_p-values were shown in Table 6.

Table 6: Conversions of dehydration of benzaldoximes.

R	σ_p[6]	Oxime / %	Nitrile / %	Amide / %	Yield / %
NO_2	0.78	5	59	35	58
Cl	0.23	-	69	31	11
H[1]	0	-	83	17	n.d.
OMe	-0.27	-	84	16	72
NMe_2	-0.83	-	91	9	70

Analytical data

4-Nitrobenzonitrile:

^{1}H NMR (500 MHz, $CDCl_3$) *δ* [ppm] = 8.40 – 8.32 (m, 2H), 7.92 – 7.84 (m, 2H).[7]

ESI-MS: *m/z* [Fragment] = 447.2 [3M+H].

4-Chlorobenzonitrile:

^{1}H NMR (500 MHz, $CDCl_3$) *δ* [ppm] = 7.63 – 7.58 (m, 2H), 7.50 – 7.43 (m, 2H).[8]

4-Methoxybenzonitrile:

^{1}H NMR (500 MHz, $CDCl_3$) *δ* [ppm] = 7.62 – 7.57 (m, 2H), 6.98 – 6.93 (m, 2H), 3.86 (s, 3H).[8]

ESI-MS: *m/z* [Fragment] = 156.9 [M-H].[8]

4-(*N*,*N*-Dimethylamino)benzonitrile:

^{1}H NMR (500 MHz, $CDCl_3$) *δ* [ppm] = 7.50 – 7.43 (m, 2H), 6.66 – 6.61 (m, 2H), 3.04 (s, 6H).

ESI-MS: *m/z* [Fragment] = 187.0 [M+H].

Screening: dehydration of 4-nitrobenzaldoxime and *N*-Boc-L-prolinal oxime using varying acceptor nitriles

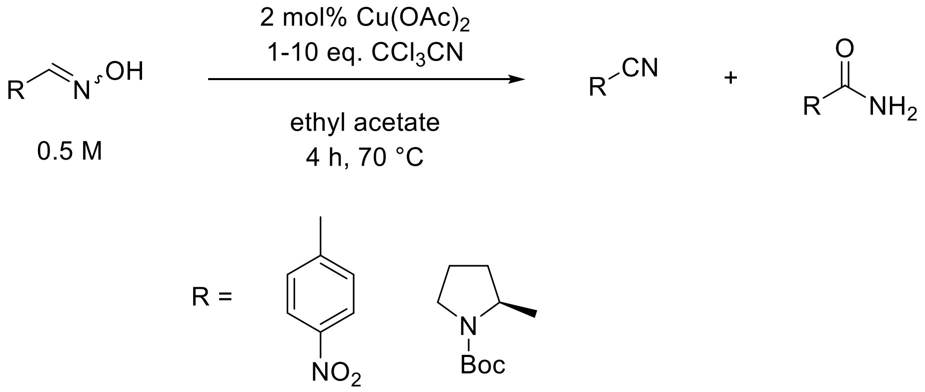

Copper(II) acetate (2 mol%) and acceptor nitrile (1, 2, 5 or 10 eq.) were added to a solution of aldoxime (1.0 eq.) in ethyl acetate (total substrate loading of 0.5 M). The solution was heated to 70 °C and stirred for 4 h. An aliquot (50 µL) was taken and analyzed via ^{1}H-NMR spectroscopy.

Table 7: Conversions of dehydration using varying acceptor nitriles.

substrate	acceptor nitrile	eq.	Aldehyde / %	Oxime / %	Nitrile / %	Amide / %	Yield / %
NO_2	CCl_3CN	10	<1	-	>99	-	n.d.
		5	<1	-	>99	-	69

		2	5	-	95	-	89
		1	<1	6	59	35	n.d.
	succinonitrile	5	<1	-	88	12	n.d.
		1	<1	4	63	33	n.d.
Boc-Prolinal oxime	acetonitrile				76	24	n.d.
	acetonitrile (dry)				81	19	n.d.
	octanenitrile		<1		74	26	n.d.
	butyronitrile		<1		73	27	n.d.
	CCl_3CN	10	<1	-	>99	-	n.d.
		2[a]	<1	-	>99	-	92

[a] reaction time: 3 h.

One-pot process with 0.1 M substrate loading in acetonitrile

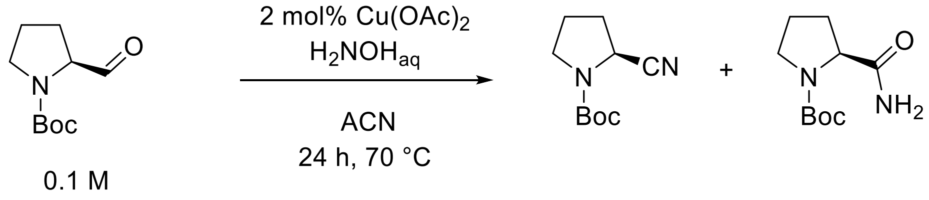

Copper(II) acetate (2 mol%, 0.8 mg, 4.6 µmol) and an aqueous solution of hydroxylamine (50 w%, 1.2 eq., 199 mg, 6.02 mmol, 398 µL) were added to solution of *N*-Boc-L-prolinal oxime (1.0 eq., 1.00 g, 5.02 mmol) in acetonitrile (50.2 mL). The mixture was stirred for 24 h at 70 °C. An aliquot (100 µL) was taken from the reaction solution, filtrated over silica and analyzed via GC. Nearly full conversion of the oxime was observed (95% nitrile, 1% oxime, 4% amide). The solvent was evaporated and the green crude product purified by filtration over silica (cyclohexane/ethyl acetate 2:1). After evaporation of the solvent, *N*-Boc-2-(*S*)-cyanopyrrolidine was obtained as a colorless oil (835 mg, 4.25 mmol, 85%).

[α] = -98.3.

3. Spectroscopic Data

N-Boc-L-prolinal oxime

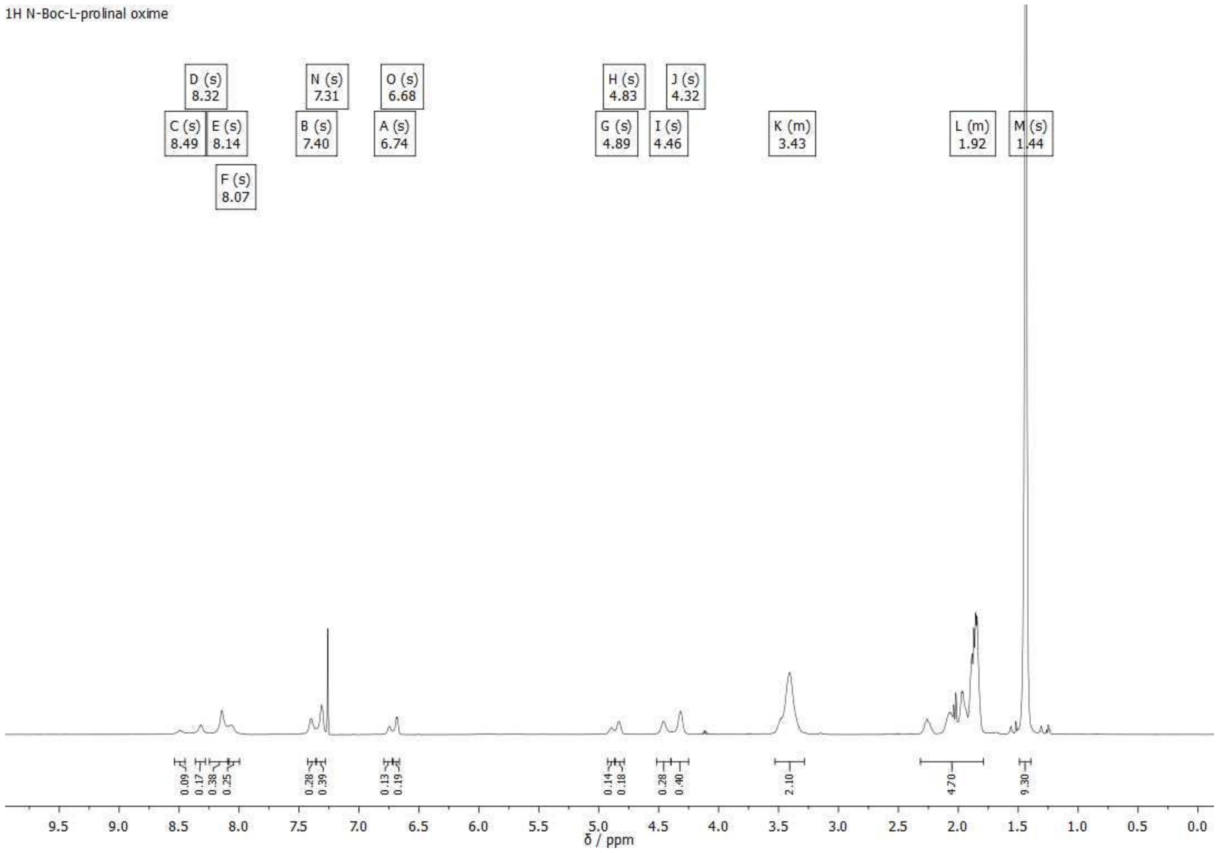

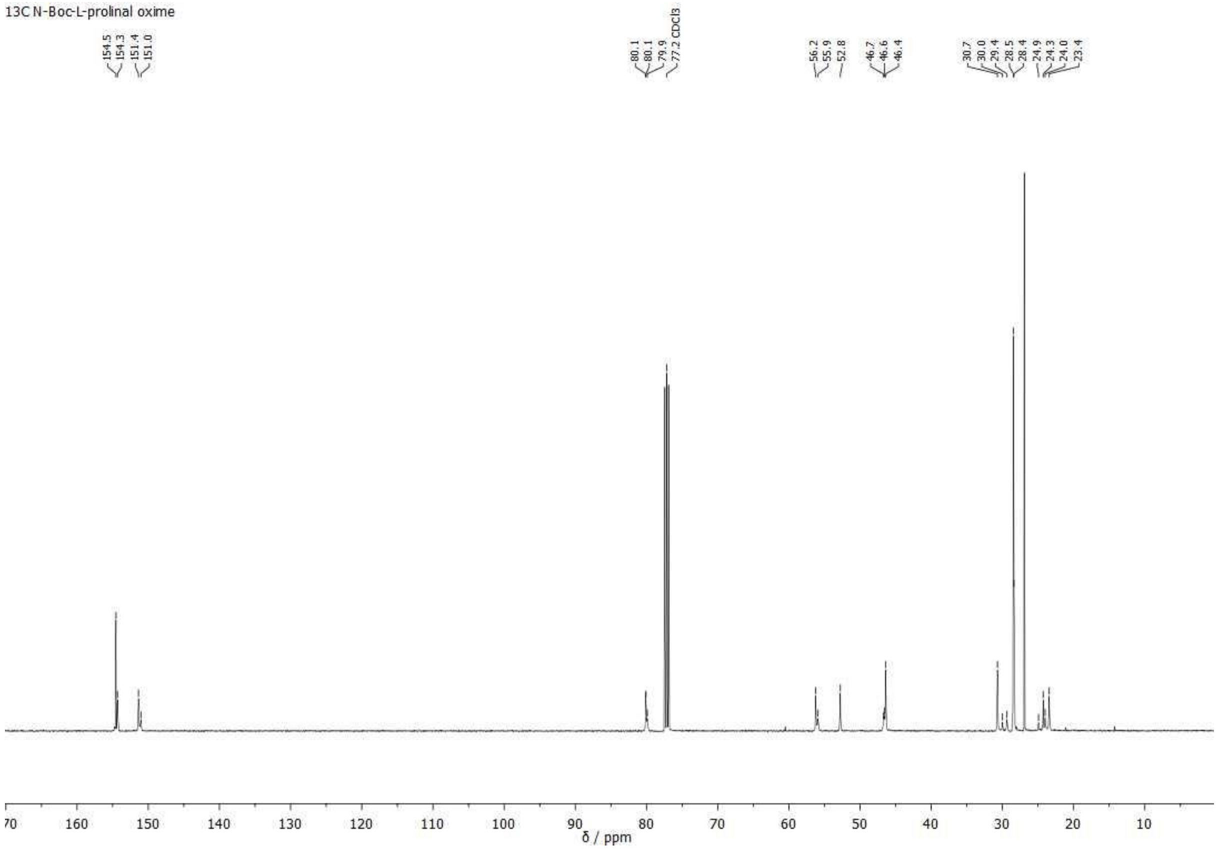

N-Boc-(*S*)-2-cyanopyrrolidine

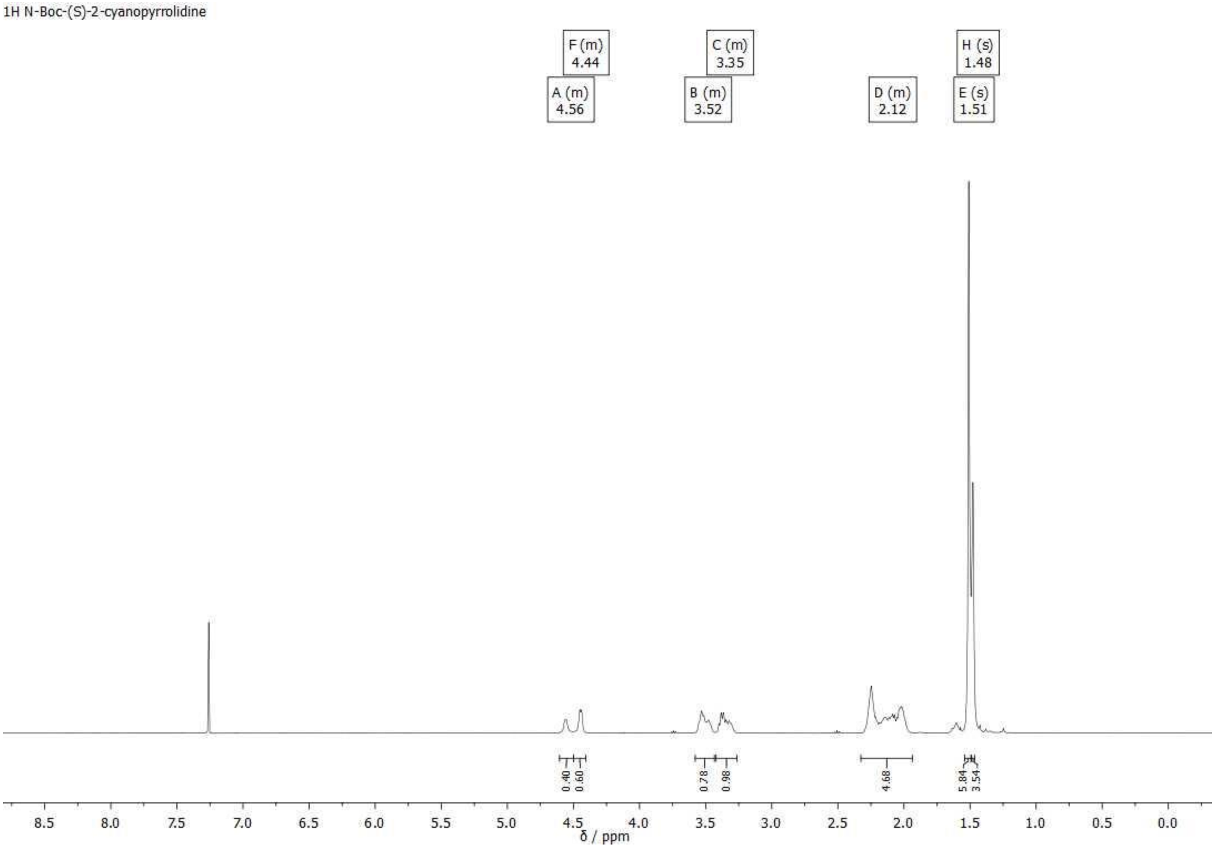

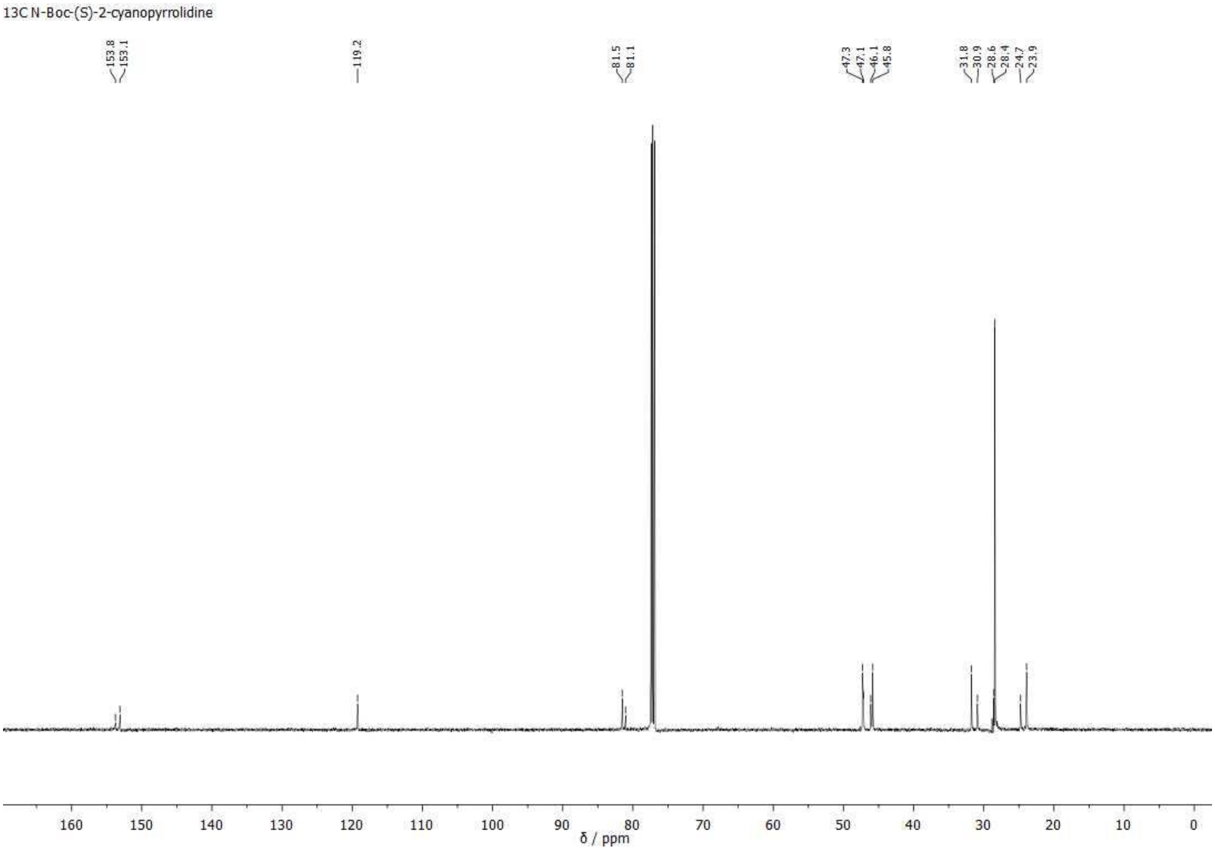

4. References

[1] W. Xu, J. Wang, C. Liu, C.-L. Chen, *J. Chin. Chem. Soc.* **2004**, *51*, 1259.

[2] B. C. Sanders, F. Friscourt, P. A. Ledin, N. E. Mbua, S. Arumugam, J. Guo, T. J. Boltje, V. V. Popik, G.-J. Boons, *J. Am. Chem. Soc.* **2011**, *133*, 949.

[3] J.-T. Li, Y.-X. Chen, X.-L. Li, H.-J. Deng, *Asian J. Chem.* **2007**, 2236.

[4] S. K. Sharma, S. D. Bishopp, C. Liana Allen, R. Lawrence, M. J. Bamford, A. A. Lapkin, P. Plucinski, R. J. Watson, J. M. Williams, *Tetrahedron Letters* **2011**, *52*, 4252.

[5] R. R. Gowda, D. Chakraborty, *Eur. J. Org. Chem.* **2011**, *2011*, 2226.

[6] C. Hansch, A. Leo, R. W. Taft, *Chem. Rev.* **1991**, *91*, 165.

[7] K. Murugesan, K. Donabauer, B. König, *Angew. Chem. Int. Ed.* **2021**, *60*, 2439.

[8] P. Anbarasan, H. Neumann, M. Beller, *Chemistry* **2010**, *16*, 4725.
